# Current treatment strategies targeting histone deacetylase inhibitors in acute lymphocytic leukemia: a systematic review

**DOI:** 10.3389/fonc.2024.1324859

**Published:** 2024-02-21

**Authors:** Yingjun Zhang, Ge Zhang, Yuefang Wang, Lei Ye, Luyun Peng, Rui Shi, Siqi Guo, Jiajing He, Hao Yang, Qingkai Dai

**Affiliations:** ^1^ Department of Laboratory Medicine, West China Second University Hospital, Sichuan University, Chengdu, China; ^2^ Key Laboratory of Birth Defects and Related Diseases of Women and Children, Sichuan University, Ministry of Education, Chengdu, Sichuan, China

**Keywords:** acute lymphocytic leukemia (ALL), histone deacetylase inhibitors (HDACi), chemotherapy, immunotherapy, targeted therapy

## Abstract

Acute lymphocytic leukemia is a hematological malignancy that primarily affects children. Long-term chemotherapy is effective, but always causes different toxic side effects. With the application of a chemotherapy-free treatment strategy, we intend to demonstrate the most recent results of using one type of epigenetic drug, histone deacetylase inhibitors, in ALL and to provide preclinical evidence for further clinical trials. In this review, we found that panobinostat (LBH589) showed positive outcomes as a monotherapy, whereas vorinostat (SAHA) was a better choice for combinatorial use. Preclinical research has identified chidamide as a potential agent for investigation in more clinical trials in the future. In conclusion, histone deacetylase inhibitors play a significant role in the chemotherapy-free landscape in cancer treatment, particularly in acute lymphocytic leukemia.

## Graphical Abstract

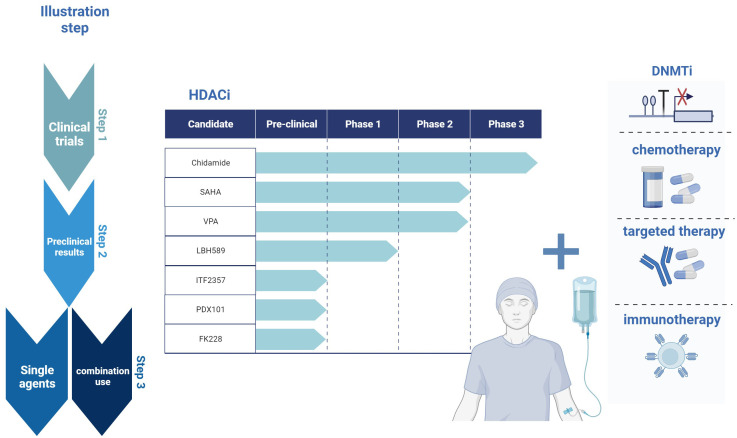

## 1 Background

Leukemia is the leading cause of pediatric malignancies, with acute lymphocytic leukemia (ALL) accounting for over 70% of cases. ALL is a rapidly invasive disease that originates from B- or T-lymphocyte progenitors. Accumulation of blast lymphocytes and suppression of normal cells are the main characteristics of the disease course. ALL predominantly affects children, with an incidence of 3–4/100,000 in patients under 14 years of age. The five-year survival rate is approximately 90% in children and 65% in adults (1). Long-term chemotherapy is the standard treatment for ALL. Common regimens include vincristine, dexamethasone, prednisone, and doxorubicin, as recommended by the American Cancer Society. However, almost all chemotherapeutic drugs cause side effects that are significant factors in clinical trials and basic research. In the last decades, other anti-cancer agents like targeted therapy and immunotherapy play a crucial role in hematological diseases. Epigenetic drugs and its various biology functions are leading topics in the treatment strategies.

### 1.1 Acetylation and deacetylation

**Figure 1:**
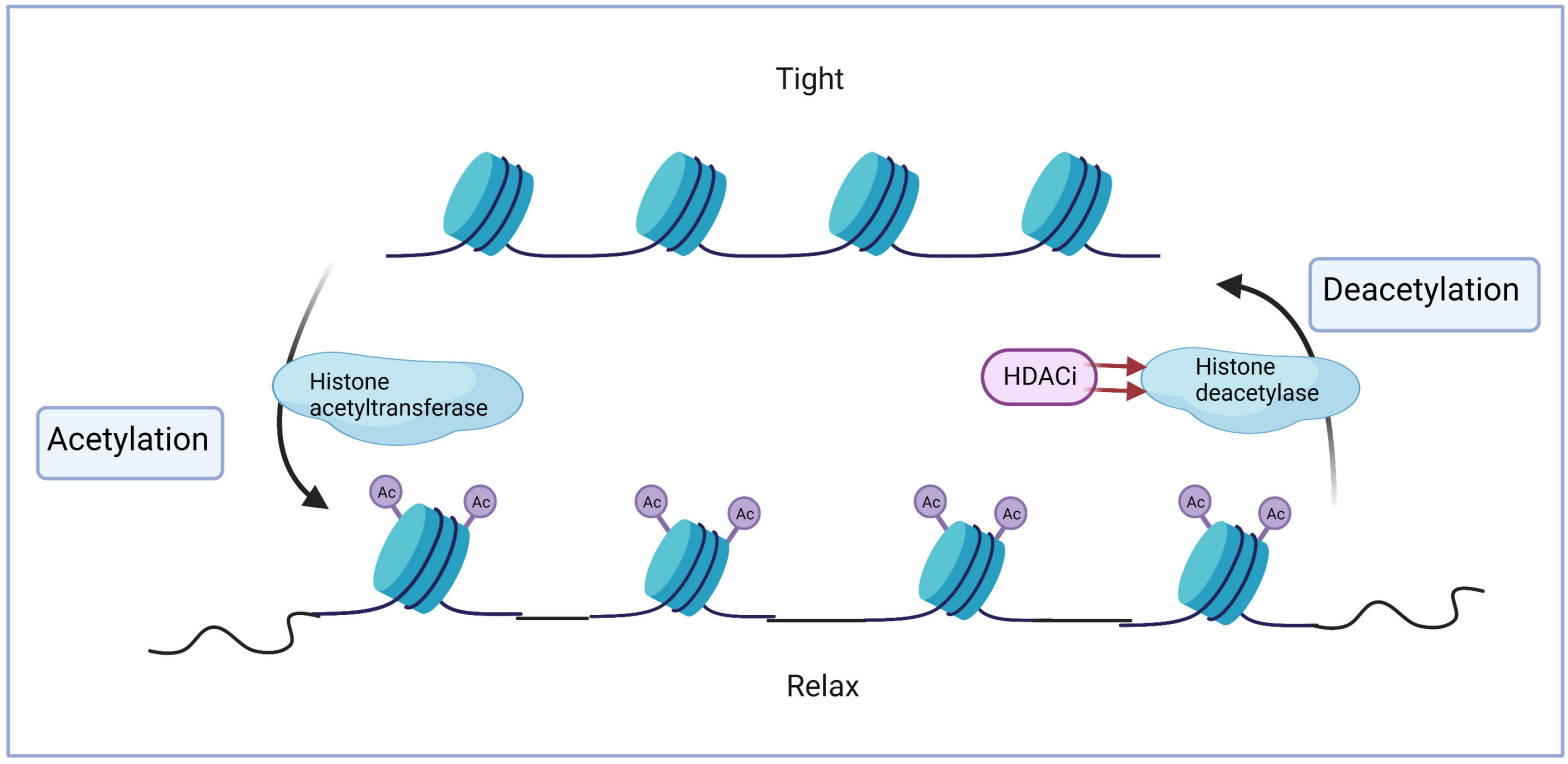
Histone acetylation and deacetylation.

Histone deacetylase inhibitors (HDACi) are epigenetic drugs that target the regulation of histone modifications. Histone acetylation and deacetylation are essential processes that regulate the chromosomal integrity. These processes are controlled by two enzymes: histone acetyltransferases (HATs) and histone deacetylases (HDACs), respectively. Acetylation can cause a loose state by neutralizing the positive charge on the surface of histones, whereas deacetylation has the opposite effect. An imbalance in this process can mediate gene expression, thereby contributing to the occurrence of diseases, where HDACi inhibit histone deacetylation and reverse the aberrant expression of specific genes (2). (**Figure 1**) Further, acetylation or acetylation on non-histone proteins has been highlighted as well in recent years, which play a role in controlling not just cancer- related transcriptional factors, but also tumor suppressor genes and oncogenes. For instance, HDAC1 directly causes deacetylation of *p*53 while HDAC2 regulates *p*53 expression via deacetylating the C-terminal lysine on p53. HDAC6 can also bind to the C-terminal region of p53 and deacetylates it (3, 4). Similarly, STAT3-phosphorylation was prevented by inhibiting the function of HDAC3 (5). HDAC6 regulates STAT3 in the same way was also proved in 2014, indicating it can be served as a novel molecular target and also a prognostic marker in B-cell lymphoma (6). In previous research targeting the aberrantly expressions of HDAC in hematologic malignancies, expression level of HDAC1-11 were detected in primary ALL patients, in which HDAC1, HDAC2 and HDAC8 were higher expressed in investigated samples and HDAC4 can be used as a therapeutic target (7).Upregulation of HDAC1-3 were found in Hodgkin’s lymphoma, whereas HDAC1-2 and HDAC6 were overexpressed in diffuse large B-cell lymphoma as well (8).

### 1.2 Classification of HDACi

**Figure 2:**
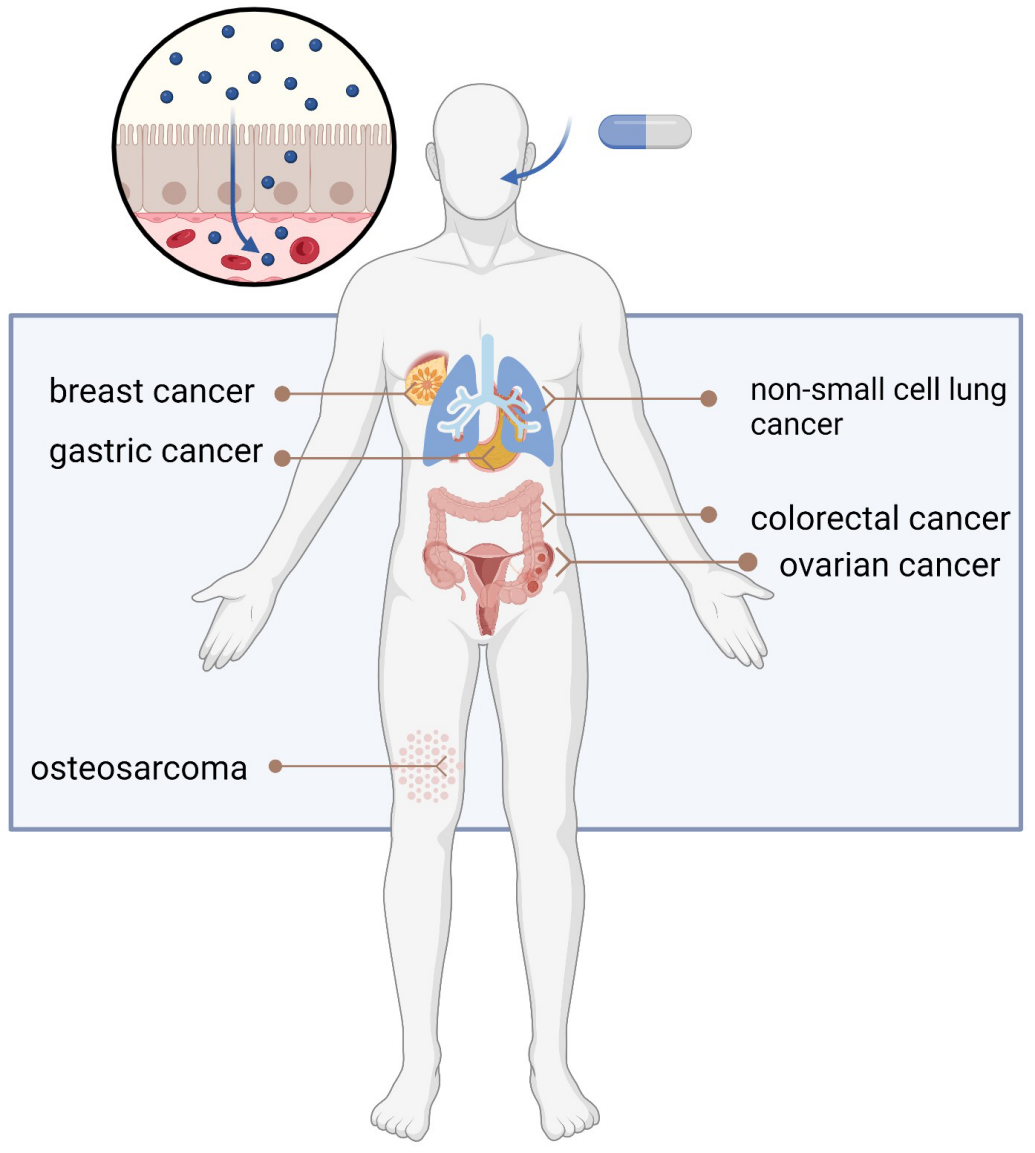
Appliance of HDACi in different cancer types.

Despite the irreplaceable role of chemotherapeutic drugs in cancer treatment, HDACi have been demonstrated to be effective against many cancer types when used alone or in combination with other drugs (**Figure 2**) (9–14). Currently there are over twenty HDACi which can be divided into pan-HDACi and specific HDACi based on their target on different classes of HDACs. HDACs was categorized as Class I (HDAC 1, 2, 3, and 8), ClassIIa (HDAC4,5,7 and 9), ClassIIb (HDAC6 and 10), Class IV (HDAC11) and Sirtuins III (SIRT 1, 2, 3, 4, 5, 6 and 7) by Zn^2+^ dependent and NAD^+^ dependent for deacetylation activity. The majority of HDACi are pan-HDACi which block more than one class of HDACs, while specific-HDACi only focus one class HDAC (15). Starting from 2006, the first HDACi was approved by Food and Drug Administration (FDA) for cutaneous T-cell lymphoma (CTCL), currently four HDACi have been approved for cancer treatment (**Table 1**), but none of them can be applied to leukemia.

**Table 1:** FDA approved HDACi in cancer treatment.

Year	HDACi approved by FDA	disease	targets	HDACi approved by FDA for ALL
2006	Vorinostat (SAHA)	CTCL	class I, II and IV HDACs	None
2009	Romidepsin (FK-228)	CTCL	specific class I HDACs	
2014	Belinostat (PXD101)	PTCL	class I, II, and IV HDACs	
2015	Panobinostat (LBH589)	MM	class I, II, and IV HDACs	

Given the potential effects of HDACi in the treatment of hematological malignancies and the promising effects observed in acute myeloid leukemia (AML) cases, we aimed to discuss the latest outcomes, starting from ongoing clinical trials to preclinical research on the use of HDACi in ALL, with the goal of identifying further directions for optimizing strategies for ALL treatment.

## 2 Materials and methods

A comprehensive literature search was conducted via PubMed for suitable studies published until July 31, 2023. The keywords for searches include HDACi, ALL, chemotherapy, synergy effects and targeted therapy.

## 3 Results

### 3.1 Outcomes from clinical trials in ALL involved in HDACi

In 2009, a phase I study conducted by DeAngelo et al. successfully demonstrated the positive effects of the HDACi panobinostat (LBH589) when used in combination with chemotherapy for AML treatment, achieving the overall response rate (ORR) being 60.9% and one year survival rate being 78.3% (16). However, the role of HDACi in ALL remains unclear. Currently, there are approximately ten trials investigating HDACi in ALL that provide basic information on the clinical treatment of ALL (**Table 2**).

**Table 2:** Clinical trials of HDACi involved in ALL treatment.

Year	Trial no.	Treatment strategy	Number of patients
2022	NCT03564470	A phase II/III study of **Chidamide + dasatinib** in adult Ph-like ALL	120
2021	NCT05075681	A phase I/II study of **Chidamide** + **Ruxolitinib** in T-ALL	50
2020	NCT01483690	A pilot study of **SAHA and decitabine** with chemotherapy in relapsed ALL	23
2019	NCT02518750	A phase II study of re-induction therapy in relapsed pediatric T-Cell acute lymphoblastic leukemia or lymphoma	3
2018	NCT03553238	A phase II/III study of **Chidamide** targeted therapy in adult ETP-ALL	70
2018	NCT03564704	A phase II/III study of **Chidamide** targeted therapy in adult T-LBL/ALL	80
2017	NCT00882206	A phase II study of **Decitabine and SAHA** in Relapsed Lymphoblastic Lymphoma or Acute Lymphoblastic Leukemia	15
2015	NCT01321346	A phase I study of **Panobinostat** in Children With refractory hematologic malignancies	30
2014	NCT01016990	A phase II study of **VPA** in previously treated patients with Lymphocytic Leukemia	52
2013	NCT00331513	A phase I study of **SAHA** in relapsed or refractory leukemia or myelodysplastic syndromes	40

A phase I study focused on LBH589 in children with relapsed and refractory hematological malignancies, including ALL. The results showed that LBH589 was well tolerated in pretreated pediatric leukemia patients, with no cardiac side effects, but the response to LBH589 was modest (17). Notably, a pilot study investigating the combination of SAHA and decitabine with chemotherapy in relapsed ALL was terminated due to toxicity firstly. Further, a subsequent pre-reductive study conducted by the same team demonstrated the effectiveness of this combination strategy by adjusting the doses of drugs. The study achieved an ORR of 46.2% and got the positive correlation between the methylation level and bone marrow response (18, 19).

Moreover, a phase IIb trial investigating chidamide showed significant clinical effects in relapsed or refractory adult T-cell leukemia, the ORR was 51.18% and the median progression free survival (PFS) rate was 152 days when combined with chemotherapy (20). Two phase II/III studies focusing on chidamide showed interest in subgroups such as Philadelphia chromosome (Ph)-like ALL and Early T-cell precursor (ETP)-ALL, the latest results indicate that chidamide is highly effective and well tolerated in these two subtypes, with the complete remission rate of 87% and 77% in ETP-ALL and Ph-like ALL respectively (21). Some other trials are underway currently which need further investigations. Nevertheless, there are encouraging data from preclinical research on several aspects (**Table 3**).

**Table 3:** Pre-clinical research of HDACi involved in ALL treatment.

Year	disease	treatment	Type of cell lines	Type of xenograft	pathway regulation		
					enhancement	inhibition	Others
(22)	ALL	Chidamid + Celecoxib	Nalm-6	–	PARP cleavage	**-**	G2M phase arrest, cell apoptosis
(23)	ALL	LBH589+ bortezomib	Nalm-6, REH, NB4	–	–	NF-κB	G1 phase arrest, cell apoptosis
(24)	ALL	Romidepsin+ cytarabine	–	NOD/SCID mice	DNA damage	–	–
(25)	T-ALL	LBH589	*Notch-1* T cell leukemias	–	–	*Notch-1* c-MYC	
(26)	BCP-ALL	ITF2357	MHH-CALL4, MUTZ5	mice	–	STAT5 phosphorylation	cell apoptosis
(27)	B-ALL	ITF2357	SUP-B15	–	–	BCR-ABL signal	cell apoptosis
(28)	T-ALL	SAHA+ vincristine	MOLT4	SCID mice	–	HDAC6	G2M phase arrest, cell apoptosis
(29)	T-ALL	Romidepsin	PEER, SUPT1	–	DNA hypomethylating, ROS, DNA damage, SAPK/JNK	MMP, PI3K-AKT-mTOR, Wnt/β-catenin	cell apoptosis
(30)	BCP-ALL	SAHA/VPA +Bortezomib	Reh, Nalm6, SD-1, 697, and SEM	NOD/SCID mice	*p53*, PI3K/AKT, and NF-κB	–	G0-G1 phase arrest
(31)	T-ALL	LBH589	ST1, KOB, LM-Y1, LM-Y2, KK1 and SO4	SCID mice	Acetylation of H3, H4, Caspase 2	AKT phosphorylation, Caspase 9	cell apoptosis
(32)	T-ALL	LBH589	MOLT-4	–	Acetylation of H3, H4, DNA damage	–	G2M phase arrest, cell apoptosis

### 3.2 Outcomes of preclinical stage investigations of HDACi in ALL as monotherapy

#### 3.2.1 Panobinostat (LBH589)

LBH589 is a pan-HDAC inhibitor of class I, II and IV HDACs. In the preclinical stage, LBH589 has shown positive effects on ALL both *in vitro* and *in vivo*. Gastro et al. demonstrated that LBH589 inhibited cell growth in T-ALL cell lines and effectively reduced tumor size in human ALL xenografts, providing further evidence of the clinical value of combination treatment (33).

Previous studies indicated that oncogene *Notch1*-driven transcription is the main target of LBH589 in ALL therapy (25). More than 70% of T-ALL cases present *Notch1*-activating mutations (gain-of-function), which can increase the regulation of cell cycle-related proteins by *Notch1* and thereby facilitate the proliferation of leukemia cells (34–36). Exposure to LBH589 in ALL cell lines has been shown to induce cell cycle progression, cell apoptosis, and DNA damage (32). The connection between HDACi and the *Notch* pathway was analyzed in detail. HDAC3 is recognized as a regulator of *Notch1* signaling response, and HDAC6 is another target closely related to the expression levels of *Notch3* in T-ALL (37, 38). HDACi including trichostatin A downregulate the *NAML3* pathway (39, 40).

Furthermore, LBH589 increased the survival of adult ALL xenograft models with t (4;11). LBH589 alone, as well as the combination of methotrexate (MTX) and 6MP, were investigated, but the combination groups did not show better effects than monotherapy (41). The latest results of genome-wide Clustered Regularly Interspaced Short Palindromic Repeats (CRISPR)/CRISPR-associated (Cas)9 (CRISPR/Cas9) loss-of-function screening in B-ALL cells also indicate that patients with higher *SIRT1* expression in cancer cells may benefit more from LBH589 treatment (42). These are all good hints that investigations of LBH589 are positive at the current stage, which can be further applied in clinical trials.

#### 3.2.2 Vorinostat (SAHA)

Vorinostat (SAHA) is the first FDA-approved pan-HDACi used for CTCL, targeting class I, II, and IV HDACs. SAHA has shown predominate effects in AML (43), whereas related publications on ALL conclude that SAHA is unsatisfactory as a monotherapy. The latest preclinical research suggests that acetylation of H3 and H4 is related to the drug resistance mechanism in T-ALL, and SAHA can reverse this resistance (44). SAHA also exhibits antileukemia effects in TYK2-rearranged ALL mice models (45). In general, most studies have focused on the synergistic effects of SAHA in combination with chemotherapy, which are discussed in the next section.

#### 3.2.3 Belinostat (PXD101)

Belinostat (PXD101) was approved by FDA in 2014 for the treatment of peripheral T-cell lymphoma (PTCL). Targeting class 1 and 2 HDACs increases the acetylation of H3 and H4 proteins, thereby inhibiting tumor cell growth in various cancer types such as T-cell lymphoma and breast cancer (46, 47). In the field of hematology, previous reports have demonstrated the potential effects of PDX101 on AML earlier (48, 49). The role of PDX101 in ALL has been reported as well. Interestingly, primary childhood ALL samples and several chemo-resistant ALL cell lines are sensitive to PDX101 through the apoptotic pathway, with even better cytotoxic effects than dexamethasone (50).

#### 3.2.4 Romidepsin

Romidepsin is another HDACi currently available for CTCL. A case study on the clinical use of romidepsin described that it was useful for an adult patient with T-ALL refractory to hyper-CVAD, providing a valuable indication for further research in hematology (51). Mechanistically, romidepsin is associated with DNA hypermethylation, thereby increasing reactive oxygen species (ROS) and decreasing the mitochondrial membrane. These changes may trigger T cell apoptosis and DNA damage (29). In infants, romidepsin was also found to have strong efficacy in *KMT2A*-r ALL through the DNA damage pathway, and rearrangement of this gene is a specific characteristic in more than 80% of cases (24). Similar effects can also be seen in LBH589 (33).

#### 3.2.5 Givinostat (ITF2357)

Several reports have investigated givinostat (ITF2357) in preclinical stages. Previous studies have shown that ITF2357 reduces the number of blasts in target organs through cell cycle regulation and DNA repair. It has also been found to have an antileukemic effect in xenografts (52). Moreover, ITF2357 is potential to be a treatment strategy for Ph-like ALL patients. These patients typically do not respond well to targeted agents, such as tyrosine kinase inhibitors (TKI) like imatinib, but ITF2357 has been found to induce potent apoptotic effects in Ph-like ALL cell lines (27).

#### 3.2.6 Chidamide

Chidamide is a HDACi that inhibits HDAC1, HDAC2, HDAC3, and HDAC10. It has been approved by the Chinese FDA for PTCL treatment and can be used in adult patients with T-ALL in Japan (53, 54). The role of chidamide in leukemia has been extensively studied in recent years. Chidamide has shown promising effects on *IKZF1* deletion in high-risk ALL, both *in vitro* and *in vivo* (55). The antitumor effects of chidamide on ALL cells are also correlated with *Notch1-MYC* gene downregulation and this mechanism gives a support to the reduction of minimal residual disease in ALL patients (56). Recent studies have shown that chidamide can regulate CD8 + cells in T-ALL by increasing the expression of CXCL9, which promotes tumor growth in many cancer types (57–60). Moreover, chidamide promoted CAR-T cell therapy by upregulating CD22 in B-ALL (61). Chidamide demonstrated promising effects and good safety in a preliminary study in children, with an overall survival (OS) of 94.1% and event-free survival rate of 95.2% (62).

**Figure 3:**
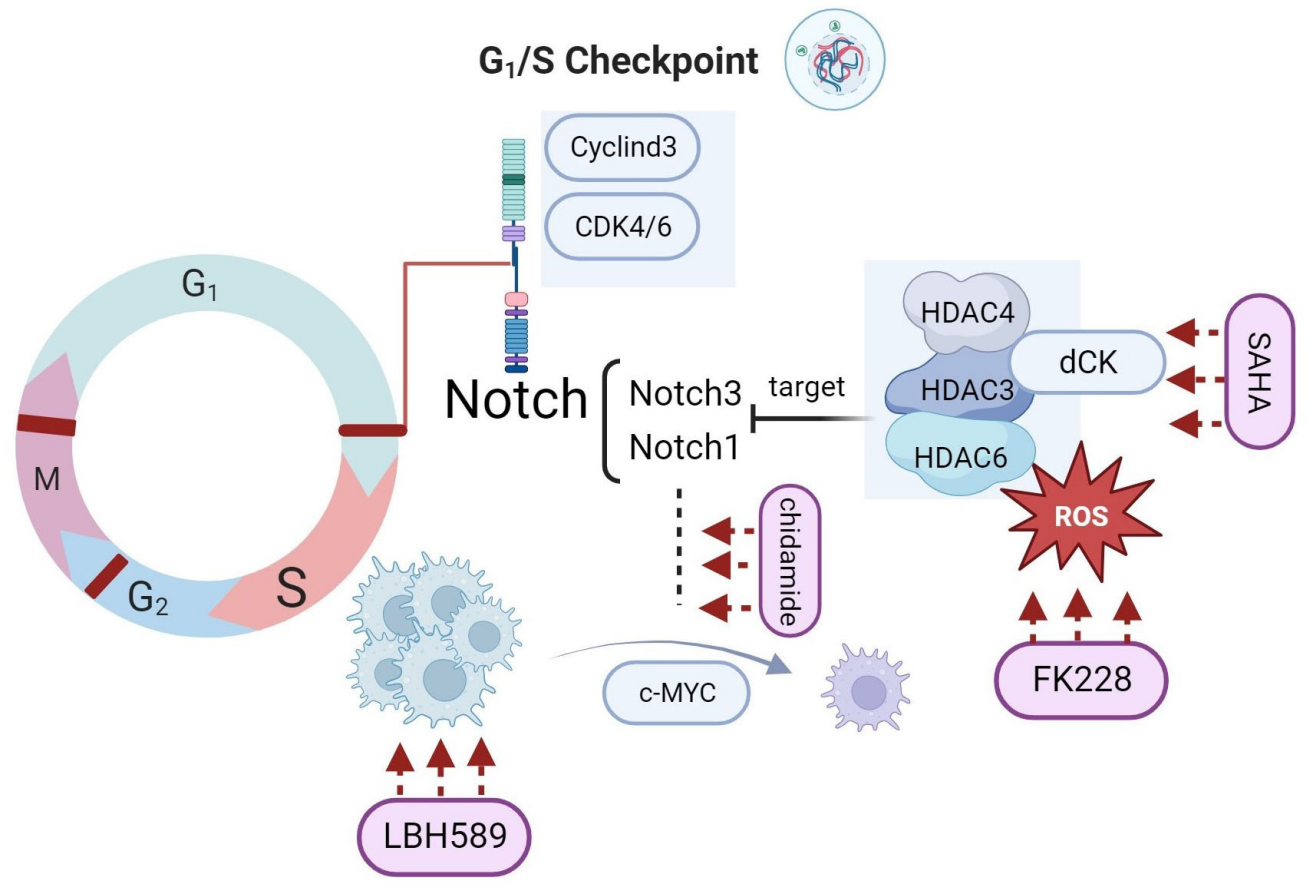
HDACi target the Notch-dependent ALL cells in different pathways.

Overall, the preclinical research of several HDACi in ALL treatment provides directions for further investigations in specific target like *Notch* signaling pathway. Correlations between agents show a closed cycle in regulations of cancers. (**Figure 3**) Moreover, some other HDACi are potential candidates for ALL based on their good effects in treating other hematological diseases (53, 63).

### 3.3 Synergistic effects of HDACi and other antileukemic agents

#### 3.3.1 HDACi in combination with other epigenetic drugs

LBH589 and azacitidine are epigenetic drugs, with LBH589 being an HDACi and azacitidine being a DNA methyltransferase inhibitor (DNMTi). The combination of these two drugs can generate synergistic effects by inducing chemoprotection in several ALL samples (64). This combination has been shown to be more efficient than using cytarabine or other drugs as monotherapy, as it can overcome the protective effects of osteoblasts on ALL cells. However, it is important to note that this specific combination has not yet been investigated in any clinical trials. Furthermore, similar strategies involving SAHA and decitabine have been tested in clinical trials and have shown promising effects in patients with relapsed ALL (18, 19, 65). Low doses of decitabine and SAHA caused cell death in leukemic cells and reduced *p21* levels without significant changes in normal peripheral blood lymphocytes (66). Additionally, pretreatment with SAHA and decitabine can enhance the cytotoxicity of chemotherapy in relapsed childhood B-ALL, suggesting that epigenetic mechanisms play a role in the acquisition of chemoresistance during ALL recurrence (67). Connective map analysis, validated by RT-PCR and gene expression arrays, identified SAHA as the most effective agent for reversing the expression of chemoresistance genes (*BIRC5*, *FOXM1*, *TYMS*, *FANCD2*, *NR3C1*, *HRK*, and *SMEK2)* in ALL relapse. Furthermore, a study reported that the combination of low-dose decitabine and chidamide enhanced apoptosis activation in adult ALL cases, particularly in patients with *p16* deletion. This enhancement was achieved by regulating *c*heckpoint kinase 1 phosphorylation and γH2A.X expression (68).

#### 3.3.2 HDACi in combination with chemotherapy

The synergistic effects of HDACi and conventional antileukemic chemotherapy drugs have been reported in several studies, in which LBH589 and SAHA have been widely discussed. In a preclinical study, LBH589 was shown to amplify vincristine's cytotoxicity in B-ALL cells (23). The study also highlighted LBH589’s ability to prolong the cell cycle and suggested that combining an autophagy inhibitor with HDACi could reverse the negative effects on nuclear factor-kappa B (NF-κB) genes in ALL treatment. Additionally, Agirre et al. demonstrated the synergistic effects of LBH589 and vincristine in xenografts of human leukemia in BALB/c-RAG2−/− γc −/− mice, and suggested that this combinatorial strategy could also be applied with dexamethasone (69). Another study by Leclerc found that SAHA enhanced the cytotoxicity of MTX in childhood ALL (70). This combination strategy has also been described for central nervous system lymphoma (71). SAHA was also shown to increase the expression of *FPGS* by 2-5-fold, while simultaneously reducing the level of DHFR and reversing its inhibitory on chemotherapy. The combination of SAHA and idarubicin is effective in pre-B-ALL, with HDAC2 implicated as a possible effector of synergism with SAHA (72). Chidamide plus chemotherapy is another strategy that has achieved better CR and ORR in patients with T-cell acute lymphoblastic lymphoma/leukemia (T-LBL/ALL) than chemotherapy alone (68). However, the underlying mechanism for this improvement remains unclear.

#### 3.3.3 HDACi in combination with targeted therapy

The clinical benefits of targeted therapeutic strategies for ALL have been observed in recent decades. According to the American Cancer Society, ALL patients with Ph+ protein show sensitivity to TKIs, such as imatinib, dasatinib, and nilotinib, particularly in B-ALL (73). While the positive effects of combining HDACi and targeted therapy have been extensively discussed in chronic lymphocytic leukemia (CLL), they have been less studied in of ALL. CLL is characterized by the aberrant accumulation of CD5+ B cells that can be targeted by HDAC6 (74, 75). Preclinical research on ALL has shown that the combination of HDACi and proteasome inhibitors such as PXD101 and bortezomib, exhibits synergistic effects through inhibiting NF-κB pathways and weakening HDAC6- mediated α-tubulin acetylation, expressions of apoptosis proteins like bim were found after the synergy use of two drugs (76). Cotreatment with a low dose of bortezomib has been found to increase apoptosis in hematological diseases (77).

#### 3.3.4 HDACi in combination with immunotherapy

Immunotherapy is a trending topic in medical oncology because it has fewer toxic side effects and is efficient. A reduction in immune evasion was found with a combination of epigenetic agents and immunotherapy, and the positive influence of HDACi on immune cells has been demonstrated in other cases (78, 79). The rationale behind the synergy between HDACi and immunotherapy can be attributed to several key factors. First, epigenetic modifications like HDACi and DNMTi can regulate T helper cells (Th) Th1 and Th2 through interferon-γ (IFN-γ). This connection was also supported by another study showing that the HDAC-Sin3A complex inhibits the accumulation of H4 acetylation by recruiting to *IFNg*-locus in Th0 cells, whereas the Th1 differential causes the loss of HDAC-Sin3A (80). Moreover, HDACs play a crucial role in regulating the functions of various immune cells such as neutrophils, eosinophils, and mast cells (81). Currently, most studies have focused on the combination of HDACi and programmed death-ligand 1(PD-L1). HDACi is a PD-L1 enhancer that inhibits tumor growth and helps overcome PD-L1 antibody resistance (82–84). In hematology, a combination of an anti-PD-1 antibody called sintilimab and chidamide has shown synergistic effects in newly diagnosed extranidal natural killer/T-cell lymphoma with minimal toxicity (85). The same combination was also mentioned by Chen et al. in a patient with relapsed/refractory transformed diffuse large B-cell lymphoma who was primarily refractory to R2-CHOP, R2-MTX, and Gemox regimens (86). Song et al. reported the first successful use of this combination strategy in the maintenance therapy of T-ALL. Bone marrow evaluation and minimal residual disease detection implied complete remission after the two-year term therapy (68). HDACi are also helpful in leukemia treatment after CAR-T cell therapy (87, 88). CD20 is a key factor in the interaction between HDACi and CAR-T cells. Reports reveal that HDACi including VPA, romidepsin could increase the expression levels of CD20 CAR-T cells as well as CAR-NK cells in cancer cells, leading to increased production of IFN-γ and tumor necrosis factor (TNF)-α (89, 90)

**Figure 4:**
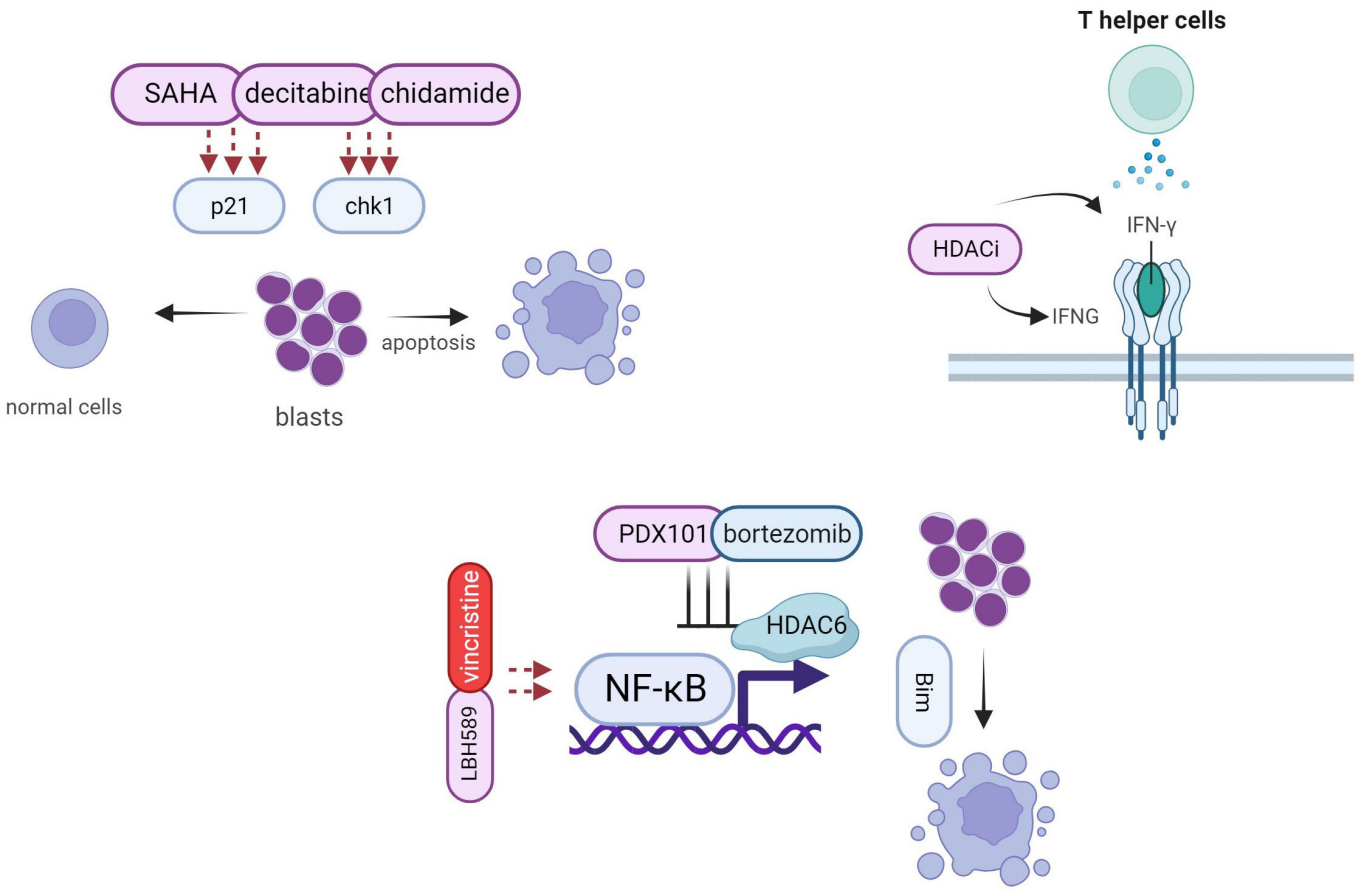
Synergy effects of HDACi and other anticancer agents.

The combination use of two types of drugs gives more chances for increasing treatment efficiency as well as discovering binding sites in two therapeutic strategies (**Figure 4**). Currently, HDACi is mainly used as an adjuvant therapy together with other types of agents, thereby promoting drug efficiency in antileukemic process.

## 4 Conclusions and prospects in different strategies

Based on previous studies, LBH589 is more important as a monotherapy than SAHA in the treatment of ALL. On the other hand, SAHA has shown potential effects mainly in co-treatment strategies. SAHA and MTX can synergize to promote ALL cell apoptosis, but lacks efficiency when combined with LBH589 (41, 70). Another strategy involving SAHA and decitabine has proven to be highly effective in both clinical trials and preclinical studies in leukemia cells (65, 91), however, very few studies have reported positive results when SAHA was used as a single agent (92). To date, numerous studies have demonstrated the effects of LBH589 as a monotherapy for ALL, providing different insights into the effects of LBH589 and SAHA. Chidamide was the first approved HDACi for T cell leukemia-lymphoma, although only in China and Japan (93). Numerous studies have investigated chidamide in the field of hematological diseases. Currently, there are 17 registered studies on ClincialTrials.gov, including 4 trials on ALL. Positive results have been widely reported in leukemia, including AML and ALL (94–97), with more cases of AML than ALL. Moreover, the role of chidamide has spread to include a maintenance effect after chemotherapy or stem cell transplantation in ALL patients (62). The efficiency of maintenance therapy after CAR-T for refractory ALL was also observed in another study (98). Considering the potential positive effects in treating ALL, further investigations on the use of chidamide as an adjuvant therapy in ALL are warranted.

In recent years, chemotherapy has led to curative outcomes but with aggressive side effects. Small molecular regimens, such as HDACi or targeted therapy, have been tried as a combinational therapeutic strategy in order to enhance the sensitivity and reduce the toxicity of chemotherapy alone. Regarding the detailed combinatorial strategies, Dieter suggested when used in combination, chemotherapy induction should be initiated in adults, followed by adolescents and finally children, as survival rates in children undergoing chemotherapy were the highest among all age groups (90%) (99). Successful treatment has been achieved in patients with aggressive non-Hodgkin lymphoma cases. Chidamide and lenalidomide resulted in complete and durable remission. Although the number of cases was limited (three patients), further evaluation of this strategy is warranted (100). Moreover, the combination of chidamide and the anti-PD-1 agent sintilimab is suitable for early-stage non-Hodgkin lymphoma (85).

In addition, toxicity issues caused by HDACi are concerns in clinics, although the side effects are less than chemo drugs. Relative toxicities have been reported in other diseases, primarily affecting the gastrointestinal, cardiac and hematology systems, in which hematological toxicities in leukemia patients should be considered carefully (101, 102). A phase I trial assessed the safety of HDACi found that the most frequent hematological effects include thrombocytopenia (28% grade 1-2; 10% grade 3-4) and neutropenia (20% grade 1-2; 16% grade 3-4) (103). Safety and tolerability should be evaluated in further clinical trials by adjusting dose of HDACi. Besides, long term toxicity indicates the epigenic modifications may promote the oncogene expression and a risk of second malignancy (104).

In conclusion, above results indicate that HDACi are promising factors in combinatorial treatment for ALL patients. Furthermore, individualized treatment should be tried in specific targets in patients with different gene mutations. Only using appropriate strategies can maximize the treatment efficiency and thereby increasing the life quality of ALL patients.

## Author contributions

YZ: Writing – original draft, Writing – review & editing, Data curation, Formal Analysis, Investigation, Methodology, Software, Supervision. GZ: Data curation, Methodology, Writing – original draft. YW: Methodology, Data curation, Writing – original draft. LY: Methodology, Data curation, Writing – original draft. LP: Methodology, Data curation, Writing – original draft. RS: Data curation, Methodology, Writing – original draft. SG: Data curation, Methodology, Writing – original draft. JH: Data curation, Methodology, Writing – original draft. HY: Data curation, Methodology, Writing – original draft. QD: Investigation, Software, Supervision, Writing – review & editing.
